# Exploring in vivo and in vitro models for heart failure with biomarker insights: a review

**DOI:** 10.1186/s43044-024-00568-1

**Published:** 2024-10-21

**Authors:** Anil Kumar Prajapati, Gaurang Shah

**Affiliations:** 1 Pharmacology Department, L. M. College of Pharmacy, Ahmedabad, Gujarat 380009 India; 2 Research Scholar, Gujarat Technological University, Ahmedabad, Gujarat 382424 India

**Keywords:** In vivo model, In vitro model, Cell line, Heart failure

## Abstract

**Background:**

Heart failure (HF) is a condition characterized by the heart’s inability to meet the body’s demands, resulting in various complications. Two primary types of HF exist, namely HF with preserved left ventricular ejection fraction (LVEF) and HF reduced with LVEF. The progression of HF involves compensatory mechanisms such as cardiac hypertrophy, fibrosis, and alterations in gene expression. Pressure overload and volume overload are common etiologies of HF, with pressure overload often stemming from conditions like hypertension, leading to left ventricular hypertrophy and fibrosis. In contrast, volume overload can arise from chronic valvular regurgitant disease, also inducing left ventricular hypertrophy.

**Main body:**

In vitro cell culture techniques serve as vital tools in studying HF pathophysiology, allowing researchers to investigate cellular responses and potential therapeutic targets. Additionally, biomarkers, measurable biological characteristics, play a crucial role in diagnosing and predicting HF. Some notable biomarkers include adrenomedullin, B-type natriuretic peptide, copeptin, galectin-3, interleukin-6, matrix metalloproteinases (MMPs), midregional pro-atrial natriuretic peptide, myostatin, procollagen type I C-terminal propeptide, procollagen type III N-terminal propeptide and tissue inhibitors of metalloproteinases (TIMPs). These biomarkers aid in HF diagnosis, assessing its severity, and monitoring treatment response, contributing to a deeper understanding of the disease and potentially leading to improved management strategies and outcomes.

**Conclusions:**

This review provides comprehensive insights into various in vivo models of HF, commonly utilized cell lines in HF research, and pivotal biomarkers with diagnostic relevance for HF. By synthesizing this information, researchers gain valuable resources to further explore HF pathogenesis, identify novel therapeutic targets, and enhance diagnostic and prognostic approaches.

## Background

### Introduction of heart failure

Heart failure (HF) is when the heart does not function properly to meet the body’s needs under normal filling pressures. Initially, the body tries to compensate with short-term adjustments involving the nervous system, hormones, and blood flow, but these adaptations lead to harmful consequences over time. These consequences include higher blood volume, elevated resistance in both the body’s and lung’s blood vessels, and reduced heart’s ability to contract effectively. These factors contribute to excess fluid retention, causing difficulty breathing and increased tiredness. Heart failure (HF) can be categorized into two main types: HF with preserved left ventricular ejection fraction (LVEF) and HF reduced with LVEF. The 2016 ESC HF guidelines introduced a distinct category called HF with a mid-range ejection fraction (HFmrEF), characterized by an ejection fraction of 40 to 49%, to encourage research in this intermediate range, but according to the 2021 ESC Guidelines, “heart failure with mid-range ejection fraction” has been updated to “heart failure with mildly reduced ejection fraction” (HFmrEF) [1]. This area has been less studied than heart failure with reduced ejection fraction (HFrEF, EF below 40%) and heart failure with preserved ejection fraction (HFpEF, EF 50% or higher). Heart failure with mildly reduced ejection fraction (HFmrEF) accounts for 10–25% of the total heart failure population. Heart failure with mildly reduced ejection fraction (HFmrEF) exhibits traits common to both heart failure with preserved ejection fraction (HFpEF) and heart failure with reduced ejection fraction (HFrEF). However, in some respects, particularly the high prevalence of ischemic heart disease, HFmrEF is more closely aligned with HFrEF. The levels of NT-proBNP in HFmrEF are similar to those in the HFpEF group but lower than those in the HFrEF group. [2, 3]. Dysfunction of the heart can arise from and sustain a variety of compensatory mechanisms. These mechanisms include the enlargement of heart muscle cells (cardiomyocyte hypertrophy), the development of fibrous tissue and cell death within the heart, the expansion of the heart’s chambers, increased heart rate (tachycardia), narrowing of blood vessels in the body’s periphery (peripheral vasoconstriction), and alterations in how the body regulates sodium and water balance, also kidney glomerular filtration [4]. The development of cardiac enlargement and fibrosis eventually leads to the onset of heart failure [5]. Every year, approximately 18 million people lose their lives due to heart problems, and this is according to recent statistics from the World Health Organization (WHO). These deaths make up 39% of male deaths and 45% of female deaths. In high-income countries, it costs an enormous 7.4 billion Yen each year to save lives from cardiovascular diseases (CVDs). Globally, the situation is getting worse. For example, in the USA, the costs of CVD are expected to rise sharply, from $555 billion in 2015 to a staggering $1.1 trillion by 2035 [6, 7]. Ischemic heart disease (IHD), characterized by insufficient blood supply to the myocardium due to coronary artery blockages or dysfunction, serves as a leading cause of morbidity and mortality on a global scale. The condition presents various symptoms and signs indicative of compromised cardiac function, including fatigue, exercise intolerance, dyspnea, and fluid retention, reflecting the heart’s inability to adequately meet metabolic demands [8]. As a leading cause of HF, IHD encompasses conditions such as coronary artery disease (CAD) and myocardial infarction (MI), which significantly elevate the risk of developing HF. Incidence and prevalence rates of HF in IHD vary among populations and are influenced by factors such as age, gender, comorbidities, and healthcare accessibility [9]. Ischemic insults trigger adverse remodeling processes, compromising contractile function and promoting ventricular dilation, contributing to the development of HF. Diabetic cardiomyopathy (D-CMP) is a type of heart condition linked to diabetes mellitus (DM), leading to notable changes in the morphology and physiology of the heart muscle, independent of additional cardiac risk factors like CAD and hypertension [10, 11]. Diabetic cardiomyopathy (D-CMP) is primarily characterized by disrupted energy metabolism, attributed to glucose toxicity, lipotoxicity, and mitochondrial dysfunction [12]. While normal hearts typically maintain low levels of type I, and type III collagen, TGF‑β1, diabetic hearts experience heightened stimulation from various sources including local renin–angiotensin system (RAS) activity, oxidative stress-induced inflammation, and hyperglycemia. Consequently, the level of transforming growth factor beta 1 (TGF‑β1) increases, prompting the conversion of cardiac fibroblasts into myofibroblasts and leading to excessive synthesis of collagen, fibronectin, and proteoglycans and at last causing heart failure [13]. According to the Animal Models of Diabetic Complications Consortium (AMDCC), a suitable animal model for diabetic vascular disease should demonstrate a minimum of one of the following characteristics: (1) dysmetabolic syndrome; (2) insulin resistance; (3) impaired glucose tolerance; (4) type 1 diabetes mellitus (T1DM); and (5) type 2 diabetes mellitus (T2DM) accompanied by atherosclerosis and peripheral vascular disease microvascular disease [14]. Various animal models are utilized to mimic diabetes mellitus (DM) and study its effects on cardiac and systemic functions. Among these models, the administration of streptozotocin and alloxan induces hyperglycemia in rodents, pigs, and non-human primates (NHPs) within days of injection. This hyperglycemic state reflects the diabetic condition and allows for the investigation of diabetic cardiomyopathy [15–17]. Additionally, high-fat diets rich in fat and low in carbohydrates, along with overfeeding regimens, are employed to induce type 2 diabetes mellitus (T2DM)-like conditions in rodents, zebrafish, Drosophila, pigs, and NHPs. These models emulate metabolic syndrome features, such as hyperglycemia, insulin resistance, dyslipidemia, and obesity, contributing to the development of diabetic cardiovascular complications [18–23]. Furthermore, genetically modified animal strains, including OVE26, NOD, BB, Akita, ob/ob (Lepob), db/db (Leprdb), and ZDF, serve as valuable tools to study both type 1 diabetes mellitus (T1DM) and T2DM. These models replicate various aspects of diabetic cardiac dysfunction, including alterations in cardiac structure, function, mitochondrial integrity, fibrosis, oxidative stress, and apoptosis, offering insights into the pathophysiology of diabetic heart disease [14–16]. Idiopathic cardiomyopathy (ICM) is a cardiac disorder marked by irregularities in ventricular wall thickness, ventricular cavity size, cardiac function, rhythm, and electrical conductivity, without a discernible underlying cause [24]. Idiopathic cardiomyopathy (ICM) encompasses three distinct types: hypertrophic cardiomyopathy (HCM), dilated cardiomyopathy (DCM), and restrictive cardiomyopathy (RCM). HCM is a generally hereditary heart-related condition that affects approximately 1 in 500 individuals in the general population, often leading to sudden cardiac death in young individuals. It is marked by left ventricular hypertrophy, usually accompanied by a small left ventricular cavity, along with myofibrillar disarray and diastolic dysfunction [25]. DCM is characterized by cardiac enlargement and systolic dysfunction, frequently presenting as congestive heart failure. RCM, a rare form of ICM, is distinguished by reduced ventricular filling and increased myocardial rigidity, due to diastolic dysfunction, atrial enlargement, and elevated systemic and pulmonary venous pressures [26].

## Main text

### Steps involved in heart failure

**Fig. 1:**
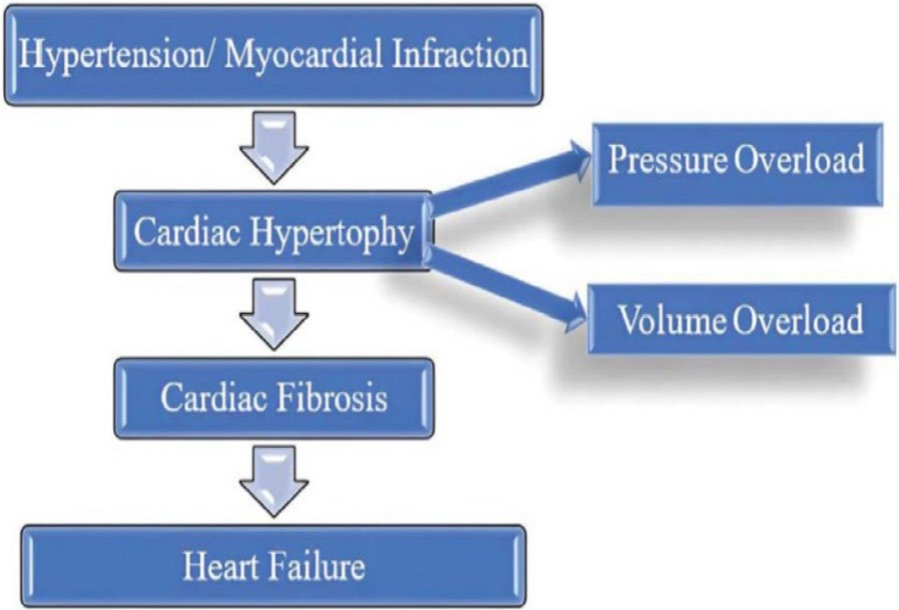
Steps involved in heart failure

Hypertension precipitates a cascade of pathological modification in the heart, culminating in cardiac hypertrophy characterized by a hypertrophy of the ventricular wall. This structural adaptation is an attempt to compensate for increased pressure load. However, the ventricles become inefficient in supplying adequate blood despite the thickened wall. Consequently, myocardial cells lack sufficient oxygen, leading to ischemic conditions and inflammatory responses. In response to ischemia and inflammation, fibroblasts within the myocardium transform into myofibroblasts. These activated myofibroblasts play a pivotal role in cardiac fibrosis, a process caused by excessive extracellular matrix (ECM) protein deposition in the cardiac tissue. The accumulation of ECM disrupts normal myocardial architecture and impairs cardiac function, ultimately contributing to the progression of heart failure (Figure 1).

### Hypertension

**Fig. 2:**
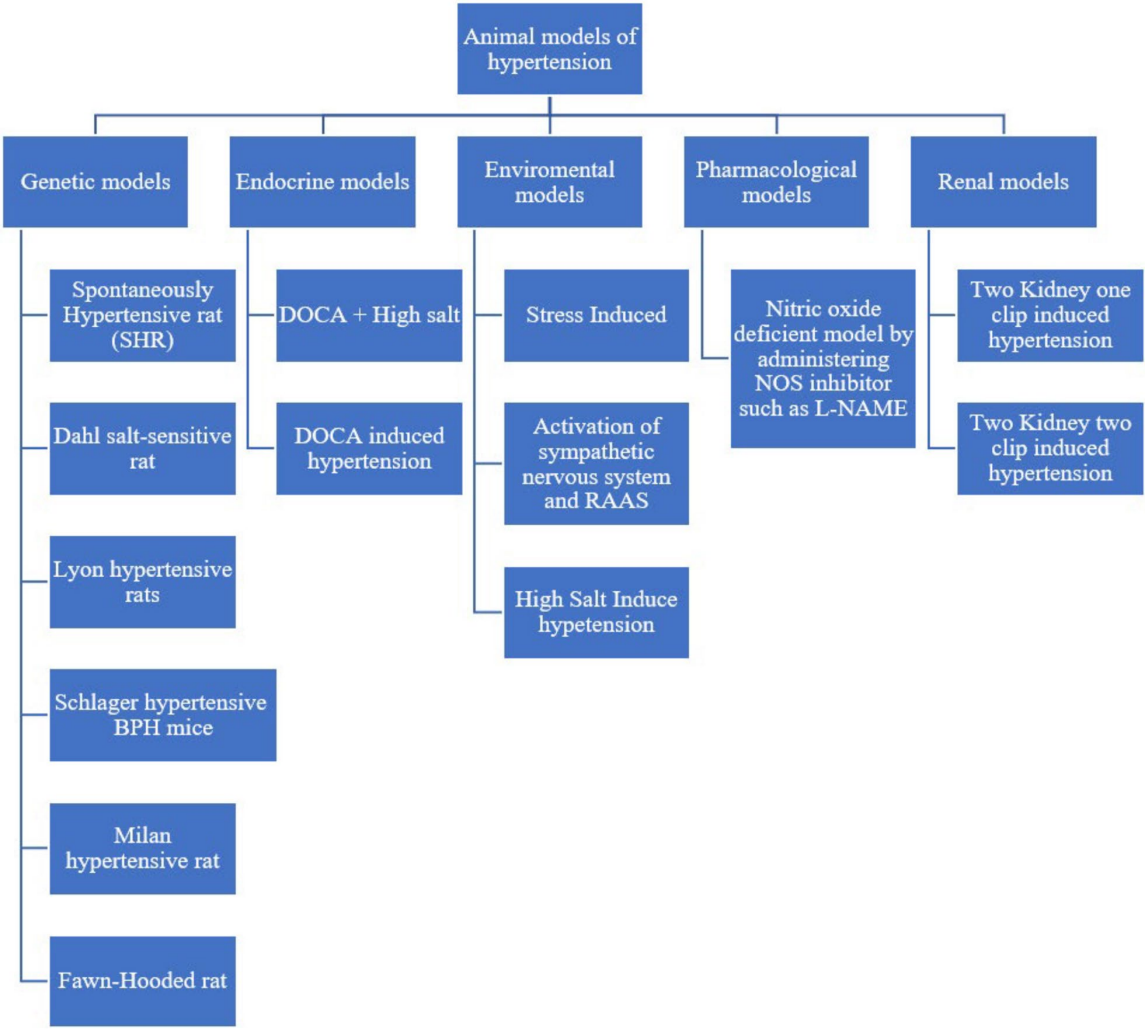
Different animal models of hypertension

High blood pressure is still a major player in causing heart failure (HF). It is associated with both the heart’s ability to pump effectively (systolic dysfunction) and relax properly (diastolic dysfunction). This disruption in the heart’s functioning involves some changes in the heart muscle, such as remodeling, making the left ventricle larger (known as left ventricular hypertrophy), and causing fibrosis [27]. Different animal models are shown in Figure 2.

### Cardiac hypertrophy

Cardiac hypertrophy, which involves the growth of heart muscle mass, represents a change in the structure of the heart muscle due to factors such as mechanical pressure and various triggers. The leading molecular factor behind this heart enlargement is the hypertrophy, or enlargement, of heart muscle cells. Although myocytes are crucial, other cell types, such as fibroblasts, smooth muscle cells, and endothelial cells, also play a role either directly or indirectly in the process of hypertrophy. The onset of hypertrophy and its progression toward heart failure is influenced by factors. These include changes in gene expression, chemical elements present in the bloodstream, cell death (apoptosis) issues with energy metabolism, irregular heart rhythm (arrhythmia) problems, and blood vessel accumulation of collagen and fibronectin levels. All these factors collectively impact the heart’s ability to relax and contract, ultimately leading to heart failure [28].

### Experimental animal models for cardiac hypertrophy

Experimental animal models for cardiac hypertrophy are invaluable tools in cardiovascular research, offering insights into the pathophysiology of hypertrophic heart diseases and potential therapeutic interventions. These models allow researchers to study the molecular mechanisms for cardiac hypertrophy, identify novel biomarkers, and evaluate the efficacy of pharmacological and genetic interventions.

- **Pressure overload**
  - *Transverse aortic constriction (TAC)* is the generally used surgical model to address pressure-overload-induced hypertrophy specifically by creating a constriction in the ascending aorta, akin to coarctation [29, 30]. In mice, the induction of mechanically driven cardiac pressure overload, cardiac hypertrophy, and heart failure is achieved through TAC. TAC is typically carried out via a conventional thoracic procedure or a minimally invasive approach, involving a small incision near the upper sternum for aortic banding. While this TAC model is used to study the genetic expression associated with the early stages of hypertrophy, it does not fully replicate the cardiac remodeling observed in humans. Aortic banding serves as a valuable model to investigate the development of left ventricular hypertrophy (LVH) in response to hemodynamic stress [28].
- **Volume overload**
- Volume overload, which is commonly seen in long-term aortic and/or mitral valvular regurgitant disease, leads to a rightward shift in the entire diastolic pressure–volume curve. This shift signifies an increase in chamber stiffness and typically coincides with the onset of concentric left ventricular hypertrophy (LVH), a condition frequently seen in ailments such as aortic stenosis, hypertension, and hypertrophic cardiomyopathy [31].
  - **Aortocaval shunt**
  - The procedure involves incising the vena cava and the abdominal aorta above the renal arteries. The aorta is then clamped just before the renal arteries. A fine needle, measuring 0.6 mm, is inserted into the aorta below the clamp, extending it into the vena cava to establish a connection between the two vessels. After this step, the needle is withdrawn, and the incision is closed. Cardiac hypertrophy typically manifests within a span of 4 to 5 weeks and is accompanied by impaired left ventricular (LV) contractility and an increase in end-diastolic pressure [31].

### Cardiac fibrosis

Cardiac fibrosis, defined by an overabundance of extracellular matrix (ECM) deposited by cardiac fibroblasts (CFs), is a prevalent pathological occurrence in numerous heart disorders such as hypertensive heart disease, myocardial infarction (MI), and various forms of cardiomyopathies. It has detrimental effects on the heart’s physical and electrical functioning [32–34]. Fibrosis arises when fibroblasts become abnormally activated and can be classified into reparative, reactive, and infiltrative. Reparative fibrosis is triggered by the loss of cardiomyocytes, leading to the formation of scars to maintain the structural integrity of the myocardium. Reactive fibrosis does not follow cardiomyocyte loss; instead, it begins when activated fibroblasts degrade the existing tissue structure and excessively deposit both fibrillar and nonfibrillar extracellular matrix (ECM) proteins, replacing cardiomyocytes [35]. Infiltrative fibrosis is defined as fibrosis caused by the buildup of non-collagenous substances such as amyloid (in the case of cardiac amyloidosis), iron (in hemochromatosis), and glycosphingolipids within the extracellular matrix (ECM) or myocytes of the heart [36]. Animal models of cardiac fibrosis and heart failure are summarized in Table 1.

**Table 1:** Animal model for cardiac fibrosis and heart failure

Model of cardiac fibrosis	Description	Degree of fibrosis*	Advantages	Limitations	References
High-salt (HS) diet	Commercial (HS) diet (4–8% NaCl)	+ to + +	1. Continual cardiac fibrosis	A mild degree of fibrosis emerged following prolonged consumption of a high-salt (HS) diet	[41, 91–96]
			2. Relevant to human fibrotic progression		
			3. Can be established in various species (rats or mice)		
			4. Can manifest independently of blood pressure changes		
			5. Impacts multiple organs within the body		
Deoxycorticosterone acetate (DOCA) salt	Unilateral nephrectomy + DOCA + HS diet (1%)	+ + +	1. Vigorous and advancing cardiac fibrosis	1. Surgical intervention is needed	[97–100]
			2. Can be induced in a variety of species, including rats and mice	2. One kidney is remaining after surgery	
			3. Associated with cardiac dysfunction and hypertension		
			4. Affects multiple organs in the body		
Dahl salt-sensitive rats (DSS rats)	Salt-sensitive SD rats + HS diet (8%)	+ + +	Sturdy and consistent cardiac fibrosis that shares similar characteristics with human HF and affects multiple organs	Time-consuming (costly)	[101–105]
Spontaneously hypertensive rat (SHR)	Hypertensive rats with a shared genetic background	+ + (adult) to + + + (aged > 12 months)	1. Consistent and naturally occurring fibrosis	Time-consuming (costly)	[106–108]
			2. Impacts multiple organs		
	Genetically related hypertensive rats				
	Gradual rise in extracellular matrix (ECM) with age		4. Associated with high blood pressure		
Spontaneously hypertensive stroke prone (SHRSP) rat	A specific subgroup of spontaneously hypertensive rats	+ + +	1. Naturally occurring fibrosis in the heart and kidneys	1. Early-onset severe high blood pressure	[109–112]
			2. Associated with high blood pressure	2. Prone to strokes	
	(SHRs) experiences the development of cerebrovascular disease				
			4. Worsened by increased salt intake	4. Uncertain survival outcomes when exposed to salt	
Ang II	Ang II subcutaneous infusion	< 1000 ng/kg/min: + to + + , ≥ 1000 ng/kg/min: + + +	1. Significant blood pressure increases and cardiac fibrosis is rapidly induced	The quick development of hypertension (and cardiac fibrosis) does not replicate the timeline seen in humans	[113–117]
			2. Uncomplicated pump implantation		
			3. Applicable to both rats and mice		
			4. Straightforward procedure		
			5. Affects various organs		
Nitric oxide synthase (NOS) inhibitors	L-NAME in drinking water or daily gavage	+ + +	1. Gradual formation of cardiac fibrosis	Insufficient proof regarding cardiac dysfunction	[118–122]
			2. Treatment without invasive procedures		
Isoprenaline	Isoprenaline injection/subcutaneous infusion	+ + to + + +	1. No surgery is required	1. Numerous diverse protocols	[123–133]
			2. Produce similar both reparative and recurring cardiac fibrosis	2. Substantial variability among different strains	
Transverse aortic constriction (TAC)	Constriction of the aortic arch	+ + +	A vigorous pressure overload model of cardiac fibrosis accompanied by cardiac dysfunction	Surgical procedures demand advanced training, and the extent of fibrosis varies with the constriction technique	[43, 134–137]
Myocardial infraction (MI)	Permanent left coronary artery ligation	+ + +	1. Replicates human heart failure-induced cardiac ischemia	Surgical procedures demand extensive training, and there is a risk of mortality associated with these surgeries	[138–141]
			2. Demonstrates significant cardiac fibrosis progression with cardiac dysfunction		
			3. Advanced techniques can minimize surgery duration and mortality rates		
Ischemia reperfusion (IR)	Transient left coronary artery ligation	+ +	Replicates human heart failure with a reperfusion component, resulting in significant cardiac fibrosis and heart dysfunction	Surgical procedures demand extensive training, with the potential for surgical complications and mortality	[142–147]

Degree of cardiac fibrosis*: + , onefold to twofold increase from control; +  + , twofold to threefold increase from control; +  +  + , fourfold or more increase from control

Animal models serve as invaluable tools for studying different aspects of heart failure, shedding light on its complex mechanisms, and facilitating the development of novel therapeutic interventions. Isoproterenol-induced heart failure replicates β-adrenergic receptor overstimulation seen in chronic stress and heart failure, providing a platform for investigating myocardial remodeling and testing β-blockers [37–39]. Angiotensin II-induced hypertension and heart failure model recapitulate renin–angiotensin–aldosterone system activation and hypertensive heart disease, aiding in understanding hypertension-induced heart failure pathophysiology and assessing renin–angiotensin–aldosterone system (RAAS) inhibitors [40–42]. The transverse aortic constriction model mimics pressure-overload-induced cardiac remodeling seen in aortic stenosis, allowing exploration of pathological hypertrophy and fibrosis mechanisms [43, 44]. Lastly, the high-salt diet model mirrors salt-induced hypertension and cardiac hypertrophy, enabling investigation of excessive salt intake effects on cardiovascular health and testing interventions targeting sodium retention and hypertension [45]. These models collectively provide invaluable insights into heart failure pathogenesis and therapeutic strategies.

### In vitro model of cardiac fibrosis and cardiac failure

The in vitro cell culture technique was developed by scientists as a suitable substitute for in vivo research in the 1800s. Cell culture has been extensively employed in academia as well as industry for a variety of purposes, from drug discovery to cell biology research. Cell culture refers to a method for nurturing cells under specific conditions such as the right growth medium, temperature, and pH levels. This technique has been in widespread use for over a century, finding applications in virus research, vaccine development, gene function studies, and pharmaceutical manufacturing. In the realm of fundamental research, cells play a pivotal role in investigating aspects of cell biology, including protein and gene functions, cellular pathways, drug safety, and the mechanisms behind various diseases. The use of cell culture offers several advantages, including accessibility, cost-effectiveness, ease of handling, and rapid results. A key reason for its popularity is the reproducibility and consistency of data generated through cell culture. This is facilitated by the uniformity of cell populations and well-established culture protocols, encompassing aspects such as growth media, subculture procedures, culture conditions, and quality control measures such as mycoplasma testing, karyotyping, confirmation of pluripotency, and differentiation [46]. Cell line/in vitro models used for cardiac fibrosis and heart failure are mentioned in Table 2.

**Table 2:** Cell lines for cardiac fibrosis and heart failure [148, 149]

Sr. no	Name of cell line	Advantages	Limitations	Applications
1	NIH 3T3 murine fibroblasts	Rapid cell growth	Limited applicability to in vivo research	DNA transfection experiments
		Convenient and straightforward handling		Investigating calcium-mediated actin reset (CaAR) triggered by physiological alterations
		Endless cell supply		Exploring the impact of transient receptor potential vanilloid 3 channel (TRPV3) activation on the differentiation of adipocytes
2	Primary rat or murine cardiac fibroblasts	Increased biological significance	Demands precise isolation and cultural techniques	Function of the human heart
		Enhanced translatability to in vivo settings, as mice or rats often serve as the initial in vitro model	Prolonged culture leads to cell differentiation into myofibroblasts	Remodeling of blood vessels
			The restricted number of passages available	Repair of myocardial tissue
				Maintenance of extracellular environment
				Providing structural support for cardiomyocytes
				Production of extracellular matrix
				Secretion of growth factors and cytokines
3	Primary human cardiac fibroblasts	Enhanced potential for application in clinical studies	Susceptible to transformation with extended culture	They contribute to scar formation post-myocardial infarction, cardiac fibrosis, and hypertrophy
		Enables the investigation of both physiological and pathological matrix remodeling	Limited availability of cells	Human cardiac fibroblasts (HCF) are valuable models for investigating these processes in vitro
			Associated with exhibit variations	
4	Immortalized human cardiac fibroblasts	Enhanced potential for translation into clinical studies	Expensive	They play a crucial role in modulating myocardial responses to injury and pathophysiological conditions, including scar formation following myocardial infarction, cardiac fibrosis, and hypertrophy. Given their responsiveness to physiological and pathological stressors, these cells serve as excellent models for studying cardiac matrix responses
		Extends the number of available passages		
		Minimizes variability in assays		
5	Neonatal cardiomyocytes (NRVMs/NMMs)	Straightforward isolation process	Juvenile phenotype	Neonatal cardiomyocytes have served as valuable tools for studying myofibrillogenesis and myofibrillar functions
		Economical		
		Offers the highest cell-to-animal ratio		
		Exhibits spontaneous beating in culture		
		Can thrive in a serum-free culture medium		
		Maintains viability for up to 28 days post-isolation		
		Small, circular cells suitable for analysis with automated cell systems (e.g., fluorescence-activated cell sorting, Coulter, etc.)		
		Responsive to hypertrophic stimuli		
6	Adult cardiomyocytes (ARVMs/AMVMs)	Economical	Isolation skills require	Drug-induced cardiotoxicity risk prediction
		Possesses a well-developed sarcomeric structure, making it ideal for patch-clamp and contractility studies	Necessitates transfection with viral vectors	Atrial fibrillation
		The presence of mature ion channels is advantageous for Ca2^ + ^imaging investigations	Limited time for maintenance in culture after isolation	Heart failure
			Lack of spontaneous beating in culture	
		Thrives in a serum-free culture medium		
		Offers access to a wide array of genetic models		
		Demonstrates a response to hypertrophic stimuli		
7	H2C9 myoblasts	Immortalized	Immature	Cardiac hypertrophy
		Uniform	Lack of spontaneous beating in culture	Cardiac fibrosis
		Rapidly expandable	Requires media supplementation with an atrial differentiation factor to induce differentiation into cardiomyocytes and express cardiomyocyte markers	
		Easily controllable		
		Originating from the ventricles		
		Responsive to hypertrophic stimuli		
8	Human embryonic and pluripotent stem cells (ESCs)	Sustained proliferation	Costly	Cardiomyocyte differentiation
		Easily controllable	Requires advanced technical skills	Myocardial repair
		Originating from the ventricles	Maintains an immature state unless cultured for 12–15 weeks	Understanding cardiac development
		Responsive to hypertrophic stimuli	Exhibits heterogeneity	
		Spontaneous contraction under optimal culture conditions		

This review was conducted without funding support

### Biomarker for heart failure

The term “biomarker,” derived from “biological marker,” was introduced in 1989 to describe a “measurable and quantifiable biological characteristic employed for evaluating the well-being and physiological condition of individuals concerning the risk and diagnosis of diseases [47].” In 2001, the National Institute of Health described a biomarker as “a trait that can be quantifiably evaluated and assessed, serving as an indication of typical biological functions, disease-related processes, or the response to medical treatment [48].” The World Health Organization further outlined a biomarker as “any substance, entity, or operation that can be quantified within the body or its byproducts and can affect or anticipate the occurrence of an outcome or disease [49].” An effective biomarker should ideally be highly sensitive and specific, and its measurement should be consistent, standardized, and economical [50].

#### Qualities of an “optimal” biomarker

A. Biomarkers must be accurately measurable.
B. The test for the biomarker should be readily accessible, cost-effective, yield swift results, exhibit minimal variability, have well-defined reference values, and its potential sources of error should be well-documented (both before, during, and after analysis).
C. The new biomarker should investigate a significant disease pathway in heart failure.
D. The substance of interest should provide insights not obtainable through physical examination or laboratory tests.
E. A new biomarker should assist in the diagnosis, risk assessment, or management of heart failure.

#### Biomarker types

A. *Preceding biomarkers* Predicting the development of future diseases.
  * Example* Myostatin and Interleukin-6 (IL-6).
B. *Screening biomarkers* Detecting hidden or subclinical diseases.
  * Example* Midregional pro-atrial natriuretic peptide (MR-proANP) and Copeptin.
C. *Diagnostic biomarkers* Identifying clinically evident diseases.
  * Example* Brain Natriuretic Peptide (BNP) and Matrix metalloproteinases (MMPs).
D. *Staging biomarkers* Determining the severity of the disease.
  * Example* Procollagen type I C-terminal propeptide (PICP) and Procollagen type III N-terminal propeptide (PIIINP).
E. *Prognostic and therapeutic biomarkers* Predicting the course of the disease and how it responds to treatment.
  * Example* Galectin-3 and Adrenomedullin (ADM).

#### Common biomarkers that are helpful in the diagnosis of heart failure

- *B-type natriuretic peptide and N-terminal B-type natriuretic peptide* Natriuretic peptides are considered the standard indicators for diagnosing and predicting heart failure. Proteolytic cleavage of the prohormone pro-B-type natriuretic peptide (proBNP) yields similar amounts of functionally active BNP and inactive N-terminal proBNP (Nt-proBNP). These factors are directly released by the left ventricular myocardium in response to elevated end-diastolic wall stress resulting from volume and pressure overload [51]. BNP functions as a vasoactive hormone that regulates fluid balance, promotes vasodilation, and influences cardiovascular remodeling [52].
  *Translation of BNP from animal to human* Animal studies provide valuable insights into regulating and releasing BNP and NT-proBNP in response to cardiac stressors like myocardial infarction, heart failure, and hypertension. These models mimic human cardiac diseases, aiding in understanding the pathophysiological mechanisms underlying cardiovascular conditions. BNP and NT-proBNP are essential biomarkers used in clinical practice for diagnosing heart failure and distinguishing between cardiac and non-cardiac causes of shortness of breath. Elevated levels indicate myocardial stretch and dysfunction. Additionally, the quantification of BNP and NT-proBNP levels offers prognostic information in various cardiac diseases, with higher levels correlating with increased mortality and adverse cardiovascular events [53].
- *Midregional pro-atrial natriuretic peptide* Atrial natriuretic peptide (ANP) is a hormone discharged by atrial cardiomyocytes in response to increased volume and wall stress [54]. In cases of heart failure, ventricular cardiomyocytes also release ANP. The cleavage of the proANP precursor yields the more durable midregional proANP (MR-proANP). MR-proANP presents a unique option for heart failure diagnosis when compared to BNP and Nt-proBNP, although it is not currently a standard part of clinical practice. Further assessment is needed to determine its significance in predicting heart failure outcomes [50].
  *Translation of ANP from animal to human* Animal studies elucidate the regulation and release mechanisms of midregional pro-atrial natriuretic peptide (MR-proANP) in response to atrial stretch and volume overload, offering an understanding of the underlying mechanisms of atrial dysfunction, heart failure, and related cardiovascular disorders. In clinical practice, MR-proANP serves dual roles as a diagnostic and prognostic biomarker for heart failure, reflecting cardiac impairment and predicting negative consequences in patients experiencing acutely decompensated heart failure. Additionally, MR-proANP levels aid in determining the timing from the initiation of atrial fibrillation to clinical presentation [55].
- *Procollagen type I C-terminal propeptide (PICP)* serum levels reflect the active synthesis of collagen type I. Patients with heart failure caused by hypertension exhibited elevated PICP serum levels [56]. Furthermore, a correlation was identified between serum PICP levels and diastolic dysfunction and ventricular hypertrophy in hypertension patients [57]. Histopathological analysis of myocardial samples from patients with hypertrophic cardiomyopathy showed a correlation between increased serum PICP levels and confirmed cardiac fibrosis [58].
  *Translation of PICP from animal to human* Procollagen type I C-terminal propeptide (PICP) is a crucial biomarker in both animal and human models for cardiac diseases, providing valuable insights into collagen synthesis and fibrosis in the myocardium. Animal studies play a key role in understanding how PICP is regulated and expressed in response to cardiac stressors like myocardial infarction, hypertension, and heart failure, shedding light on the pathophysiological mechanisms underlying cardiac fibrosis and remodeling. These animal models serve as effective tools for mimicking human cardiac diseases and studying the dynamic changes in PICP levels throughout disease progression, aiding in unraveling the diagnostic and prognostic significance of PICP in various cardiac conditions. PICP levels are indicative of prognosis in individuals with cardiac diseases, with elevated levels being associated with adverse cardiovascular outcomes such as increased mortality, heart failure progression, and adverse cardiac remodeling [59].
- *Procollagen type III N-terminal propeptide (PIIINP)* is released into the bloodstream as a consequence of the ongoing synthesis of collagen type III during cardiac fibrosis [60]. The MESA (multi-ethnic study of atherosclerosis) demonstrated that PIIINP levels can serve as an early risk stratification marker for HFpEF in a population without evident cardiovascular disease, although this association was not observed in cases of heart failure with reduced ejection fraction [61].
  *Translation of PIIINP from animals to humans* In a rat model of myocardial infarction (MI), the administration of tanshinone IIA resulted in decreased fibrosis along with a reduction in serum PIIINP levels [62]. This suggests that PIIINP may serve as an indicator of the extent of myocardial fibrosis. Patients with serum PIIINP levels exceeding 7 pg/L exhibited a heightened risk of poor hemodynamic status, advanced clinical stage, need for heart transplantation, hyponatremia, and mortality during follow-up compared to those with lower PIIINP values. This underscores the potential of elevated serum PIIINP levels as a prognostic marker correlated with the clinical stage [63]. Furthermore, PIIINP levels exhibited a positive correlation with diastolic dysfunction, left ventricular mass index (LVMI), and relative wall thickness (RWT) in individuals who had undergone successful correction of coarctation of the aorta (CoA) with accompanying left ventricular hypertrophy [64].
- *Galectin-3* is a soluble protein that binds to β-galactosides and influences cell adhesion by interacting with extracellular matrix (ECM) proteins. It plays a significant part in the processes of heart remodeling, particularly in cardiac hypertrophy and fibrosis [65, 66]. Galectin-3 is linked to an elevated risk of heart failure in the broader population, as well as increased rates of cardiovascular and overall mortality [67].
  *Translation of Gal-3 from animals to humans* Galectin-3 (Gal-3) is crucial in heart failure (HF) by contributing significantly to cardiac ventricular remodeling. While Gal-3 expression remains minimal in a healthy heart, its levels surge in HF. Notably, in rat models of myocardial infarction, Gal-3 levels have been observed to rise, particularly in regions of the myocardium not affected by the infarction but undergoing remodeling. Further research involving the infusion of Gal-3 in rats has demonstrated a notable increase in the infiltration of macrophages and mast cells, accompanied by elevated levels of cardiac fibrosis and hypertrophy. The stimulation of Gal-3 causes to the formation of a lattice complex, which effectively traps transforming growth factor β (TGFβ) on cell surfaces, thereby prolonging the fibrotic signaling process [68]. In clinical practice, Gal-3 is extensively studied as a diagnostic and prognostic marker in heart disease. It is indicative of cardiac inflammatory responses and fibrosis, with its levels reflecting the underlying pathogenesis of HF [69].
- *Interleukin-6 (IL-6)* is a crucial inflammatory mediator released into the bloodstream in response to infections and tissue damage during acute phases [70]. The BIOSTAT CHF (Biology Study to Tailored Treatment in Chronic Heart Failure) study found that IL-6 could independently predict several key outcomes, including the combined result of all-cause mortality and unscheduled hospitalization for heart failure within 2 years, as well as overall mortality and cardiovascular and non-cardiovascular mortality [71].
  *Translation of IL-6 from animals to humans* Inflammation plays a significant role in the pathophysiology of heart failure (HF). In HF, various cells release inflammatory mediators such as interleukin-6 (IL-6), indicating activation of inflammation. IL-6 not only acts as a marker for inflammation, but also contributes to the advancement of HF by inducing systolic dysfunction, ventricular dilatation, cardiomyocyte hypertrophy, and apoptosis through various mechanisms [72]. Research has demonstrated that in rat models of heart failure induced by the ligation of the left anterior descending coronary artery, ischemia and hypoxia trigger the production of IL-6. Similarly, evidence from patients with HF shows heightened levels of IL-6 in both circulation and myocardium, with IL-6 levels in circulation being associated with the progression of HF. Inflammation-mediated ventricular remodeling is a significant contributor to the initiation and worsening of HF, with IL-6 playing a regulatory role in the inflammatory process and promoting ventricular remodeling [73].
- *Matrix metalloproteinases (MMPs)* and tissue inhibitors of metalloproteinases (TIMPs) are crucial in regulating the maintenance of the extracellular matrix (ECM) within myocardial interstitial tissue. An abnormal buildup of ECM results in the development of myocardial fibrosis. MMPs are responsible for breaking down various types of proteins in the ECM, and this process is moderated by TIMPs and other cytokines. A disruption in the balance between MMPs and TIMPs is a key factor in myocardial fibrosis. In the context of myocardial fibrosis, TIMP1 consistently exhibits elevated levels and is employed as a fibrosis biomarker [74, 75]. Importantly, the expression of MMP-9 is increased in myocardial fibroblasts but not in cardiac cells [76].
  *Translation of MMPs from animals to humans* The activity of Matrix Metalloproteinases (MMPs) rises during heart failure, as evidenced by elevated levels observed in pathological samples from heart failure patients. Similarly, following myocardial infarction, MMPs experience a significant surge shortly after the event, triggered by local cytokine activation and infiltration of inflammatory cells. In animal models of myocardial infarction, heightened levels of tumor necrosis factor (TNF) within the myocardium directly correlate with increased production of local MMP-9 and MMP-2 [77]. Post-myocardial infarction, MMP-2 levels surge due to stimulating cardiomyocytes and cardiac fibroblasts. Heart failure patients demonstrate a fourfold increase in MMP-2 expression compared to controls. In rat models, MMP-2 mRNA and protein levels elevate within 24-hour post-myocardial infarction, peaking around day 14 post-event. In rat models, MMP-2 mRNA and protein levels increase within 24 hours following myocardial infarction, reaching their highest point around day 14 post-event. Similarly, in mice, MMP-2 activity rises rapidly within 4-day post-myocardial infarction, peaks at day 7, and remains elevated until day 14. MMP-9, secreted by fibroblasts, shows heightened circulating levels from day 1 after myocardial infarction, persisting until day 7. MMP-9 functions as a unique prognostic biomarker for developing left ventricular dysfunction and prolonged survival in patients with cardiovascular disease [78].
- *Adrenomedullin (ADM)* is a peptide hormone that plays a role in cardiovascular and renal functions, as well as in maintaining fluid and sodium balance. It also has inotropic action on the cardiac. Like other natriuretic peptides, including atrial natriuretic peptide (ANP), brain natriuretic peptide (BNP), and C-natriuretic peptide, adrenomedullin (ADM) helps regulate vascular tone by acting as a strong vasodilator and maintaining endothelial health [79]. The secretion of ADM is primarily triggered by an excessive volume of fluid in the body, making it a potential biomarker for tissue congestion in heart failure [80].
  *Translation of ADM from animals to humans* Plasma adrenomedullin (ADM) concentrations rise proportionately with the severity of heart failure, evident in both diastolic and systolic heart failure cases. Research conducted in laboratory settings and live subjects indicates that various hormones and cytokines can stimulate ADM production within both cardiac and vascular tissues. Mechanical stress also contributes to increased ADM production in cardiac tissues. Low doses of ADM have been observed to enhance urination and sodium excretion, while higher doses result in a slight decrease in mean arterial blood pressure but a significant increase in cardiac output in both healthy rats and those with heart failure. Notably, heart failure rats exhibit reduced right ventricular and atrial pressures alongside increased renal plasma flow, resembling the effects seen with low-dose ADM treatment. These observations suggest that ADM may play a part in managing pressure and volume in cardiac failure, a notion supported by clinical investigations. In individuals without heart failure, ADM is present in low concentrations, typically around 13 pg/mL. However, patients with chronic heart failure show plasma ADM levels three to four times higher [81]. Elevated ADM levels in heart failure are associated with the severity of the condition and serve as a reliable indicator of lingering congestion in patients experiencing acute decompensated heart failure. Numerous studies have linked heightened ADM concentrations with adverse clinical outcomes in heart failure patients [80].
- *Copeptin* is derived from the hypothalamic hormone arginine vasopressin (AVP), also known as the anti-diuretic hormone, which plays a role in maintaining fluid and sodium balance while contributing to the regulation of plasma osmolality and arterial pressure. In cases of heart failure (HF), the secretion of AVP increases in response to a reduced blood volume and decreased cardiac output. AVP has a relatively brief lifespan, while its C-terminal inactive fragment, copeptin, is more stable and easier to detect. A multicenter study involving 268 heart failure patients revealed that elevated levels of copeptin were significantly linked to a higher risk of rehospitalization and mortality. Additionally, in this study, copeptin outperformed BNP and NT-proBNP in terms of its predictive capacity [82]. Similarly, in a meta-analysis that included 4473 cases of acute and chronic HF, copeptin emerged as a strong predictor of all-cause mortality, comparable to NT-proBNP [83].
  *Translation of Copeptin from animals to humans* Research involving animal models has shed light on the diverse roles of copeptin in cardiovascular functions such as fluid balance regulation, vasoconstriction, and stress response. These studies have shown that copeptin levels are associated with changes in hemodynamics during myocardial infarction or heart failure, indicating its potential as a predictive marker [84]. Human studies have further investigated copeptin’s diagnostic and predictive significance in various cardiac conditions. Increased levels of copeptin have been linked to the severity of heart failure, the degree of myocardial injury post-acute myocardial infarction, and the likelihood of negative outcomes in individuals with cardiovascular diseases [85].
- *Myostatin (MSTN)* is a signaling molecule within the extracellular matrix (ECM) that is functionally regarded as a negative regulator of muscle mass [86]. MSTN serum levels have demonstrated predictive potential for assessing the severity and clinical progression of heart failure (HF). Patients with chronic heart failure (CHF) exhibit elevated levels of MSTN in their serum. Furthermore, increased serum MSTN levels in CHF patients are significantly associated with a reduced survival rate and a higher frequency of rehospitalizations [87, 88].
  *Translation of Myostatin from animals to humans* Increased levels of myocardial myostatin were observed in animal models following pathological stimulation. For example, in a rat model of volume-overload heart failure induced by an aortocaval shunt, there was approximately a threefold increase in myostatin mRNA and protein expression. Similarly, genetically engineered mice develop cardiac hypertrophy due to prolonged activation of Akt exhibited an 18.4-fold upregulation in myostatin mRNA levels [89]. In a study conducted by Chen et al [87], 288 hospitalized individuals diagnosed with chronic heart failure were examined. It was noted that serum myostatin concentration gradually increased with the severity of the disease, peaking in class IV patients according to the New York Heart Association (NYHA) criteria. During a 51-month monitoring period, individuals with heightened serum myostatin levels demonstrated increased rates of mortality and hospitalization. This led to the conclusion that serum myostatin concentration could independently forecast survival outcomes in patients with congestive heart failure (CHF) [90].

## Conclusion

In conclusion, heart failure represents a multifaceted and challenging condition with diverse etiologies and pathophysiological mechanisms. From ischemic heart disease to diabetic cardiomyopathy, and idiopathic cardiomyopathy, various factors contribute to its onset and progression. Animal models, such as those induced by ischemia, hypertension, or genetic manipulation, serve as invaluable tools for unraveling the intricate complexities of heart failure pathogenesis. Moreover, in vitro cell culture techniques provide complementary insights into cardiac fibrosis and dysfunction, offering a controlled environment for studying cellular responses and molecular mechanisms. Together, these approaches foster a deeper understanding of heart failure and lay the groundwork for innovative therapeutic strategies. In parallel, biomarkers emerge as indispensable tools for diagnosing, prognosticating, and managing heart failure, offering clinicians valuable insights into disease progression and treatment response. Through accurate measurement and assessment, biomarkers empower clinicians to tailor interventions and optimize patient care. Overall, the synergy between animal models, in vitro studies, and biomarker research constitutes a dynamic framework for advancing our understanding of heart failure and translating discoveries into clinical practice. By harnessing the collective power of these approaches, we can strive toward improved outcomes and enhanced quality of life for individuals affected by heart failure.

## Data Availability

This article contains no datasets generated or analyzed during the current study.

## Abbreviations

ADM: Adrenomedullin
ANP: Atrial natriuretic peptide
Ang II: Angiotensin II
CF: Cardiac fibroblasts
CVD: Cardiovascular diseases
DOCA: Deoxy corticosterone acetate
ECM: Extracellular matrix
HF: Heart failure
HFpEF: Heart failure with preserved ejection fraction
HS: High salt
IL-6: Interleukin-6
IR: Ischemia–reperfusion
L-NAME: N-nitro-L-arginine methyl ester
LV: Left ventricular
LVH: Left ventricular hypertrophy
LVEF: Left ventricular ejection fraction
MMP: Matrix metalloproteinases
MI: Myocardial infarction
MR-proANP: Midregional pro-atrial natriuretic peptide
MSTN: Myostatin
NOS: Nitric oxide synthase
Nt-proBNP: N-terminal ProBNP
PICP: Procollagen type I C-terminal propeptide
PIIINP: Procollagen type III N-terminal propeptide
RAAS: Renin–angiotensin–aldosterone system
SHR: Spontaneously hypertensive
SHRSP: Spontaneously hypertensive stroke prone
TIMP: Tissue inhibitors of metalloproteinases
TAC: Transverse aortic constriction
TNF-α: Tumor necrosis factor alpha
